# Overexpression of the riboflavin biosynthetic pathway in *Pichia pastoris*

**DOI:** 10.1186/1475-2859-7-23

**Published:** 2008-07-29

**Authors:** Hans Marx, Diethard Mattanovich, Michael Sauer

**Affiliations:** 1 Institute of Applied Microbiology, Department of Biotechnology, BOKU – University of Natural Resources and Applied Life Sciences, Muthgasse 18, 1190, Wien, Austria; 2 School of Bioengineering, FH Campus Wien – University of Applied Sciences, Muthgasse 18, 1190, Wien, Austria

## Abstract

**Background:**

High cell density cultures of *Pichia pastoris *grown on methanol tend to develop yellow colored supernatants, attributed to the release of free flavins. The potential of *P. pastoris *for flavin overproduction is therefore given, but not pronounced when the yeast is grown on glucose. The aim of this study is to characterize the relative regulatory impact of each riboflavin synthesis gene. Deeper insight into pathway control and the potential of deregulation is established by overexpression of the single genes as well as a combined deregulation of up to all six riboflavin synthesis genes.

**Results:**

Overexpression of the first gene of the riboflavin biosynthetic pathway (*RIB1*) is already sufficient to obtain yellow colonies and the accumulation of riboflavin in the supernatant of shake flask cultures growing on glucose. Sequential deregulation of all the genes, by exchange of their native promoter with the strong and constitutive glyceraldehyde-3-phosphate dehydrogenase promoter (P_*GAP*_) increases the riboflavin accumulation significantly.

**Conclusion:**

The regulation of the pathway is distributed over more than one gene. High cell density cultivations of a *P. pastoris *strain overexpressing all six *RIB *genes allow the accumulation of 175 mg/L riboflavin in the supernatant. The basis for rational engineering of riboflavin production in *P. pastoris *has thus been established.

## Background

Riboflavin, the precursor of the essential cofactors flavin mononucleotide (FMN) and flavin adenine dinucleotide (FAD), is overproduced in a variety of microorganisms. Ascomycetes like *Ashbya gossypii *(anamorph: *Eremothecium ashbyii*) [1], *Candida famata *[2] or *Pichia guilliermondii *[3] are natural overproducers of riboflavin. These, as well as other riboflavin producers have been termed flavinogenic yeasts, a phenotype which is usually induced by iron starvation [4], leading to the secretion of riboflavin in the mg/L range. After intensive strain improvement and fermentation optimization, up to 20 g/L riboflavin can be achieved today in industrial production processes, (reviewed by [2]). Published approaches to improve riboflavin production strains employ mainly random mutagenesis and screening or selection with metabolite analoga, mainly targeting at the precursers upstream of riboflavin synthesis. For the flavinogenic fungus *A. gossypii *it has been shown that the main regulation/deregulation targets are localized in guanosine triphosphate (GTP) synthesis [5], however only few details are known about transcriptional regulation of the riboflavin pathway itself [6]. In *C. famata *the deregulation targets include as well the GTP biosynthesis, but also the glycolytic flux in general [2]. However, there is still a lack of knowledge on the regulation of the riboflavin synthesis pathway and its impact on the overproduction of riboflavin.

**Figure 1:**
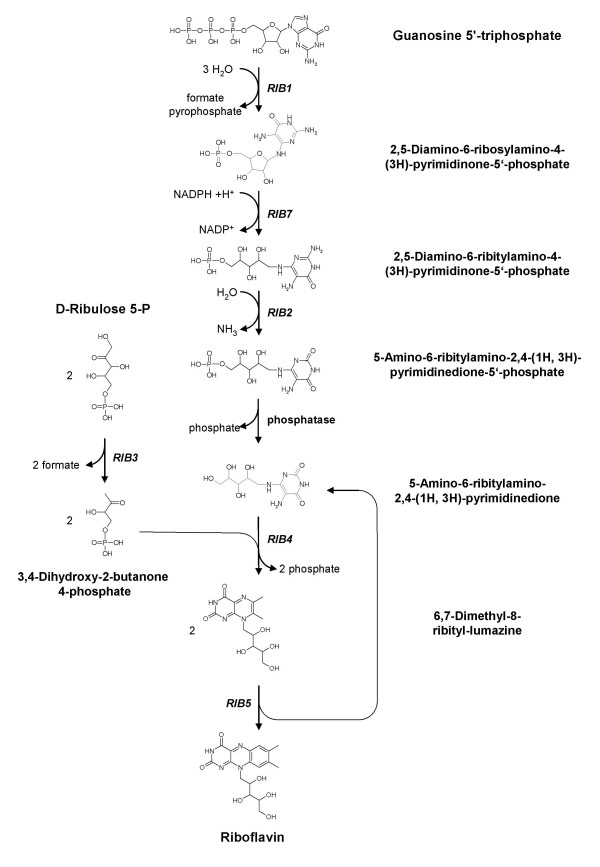
**Schematic representation of the metabolic pathway of the riboflavin biosynthesis. Gene names are following the nomenclature of the *Saccharomyces *Genome Database (SGD)**. *RIB1*: GTP cyclohydrolase II, *RIB7*: 2,5-Diamino-6-ribosylamino-4-(3H)-pyrimidinone-5'-phosphate reductase, *RIB2*: 2,5-Diamino-6-ribitylamino-4-(3H)-pyrimidinone-5'-phosphate deaminase, *RIB3*: 3,4-dihydroxy-2-butanone-4-phosphate synthase, *RIB4*: 6,7-dimethyl-8-ribityllumazine synthase, *RIB5*: riboflavine synthetase

The flavin synthesis pathway of yeasts, starting from GTP and ribulose-5-phosphate, is well established today. The pathway of *Saccharomyces cerevisiae*, and the enzymes and metabolites involved, are summarized in Figure 1. It can be assumed that other yeasts use the same pathway.

The aim of this study was to understand the relative regulatory impact of each riboflavin synthesis gene on the control of the entire pathway. Deeper insight into pathway control and the potential of deregulation can be established by overexpression of the single genes as well as combined deregulation of up to all six riboflavin synthesis genes. Interestingly, it has been shown for *Bacillus subtilis *that an increase of the *RIB *operon copy number leads to increased riboflavin production. Accordingly, it appeared attractive to investigate the impact of overexpression of single, and up to all six *RIB *genes in yeasts as well.

Methylotrophic yeasts like *Hansenula polymorpha *and *Pichia pastoris *should have a high potential of riboflavin formation considering the amount of flavin bound in alcohol oxidase (AOX). AOX, as the first step of methanol assimilation, is accumulated up to 30% of the whole cellular protein when the cells are grown on methanol as carbon source [7], so that the eight molecules of FAD in the active enzyme complex amount to 1.5 mg/g FAD per biomass under these conditions. Flavin synthesis is regarded to be highly regulated. Free FAD was shown to be the main repressor of flavin biosynthesis in *H. polymorpha *[8]. Regulation by FAD was mainly attributed to control of the genes encoding the last three enzymes in the catalytic cascade leading to riboflavin, FMN and FAD, respectively. While *P. pastoris *does not produce riboflavin when grown on glucose, high cell density cultures grown on methanol tend to develop yellow colored supernatants [9,10], attributed to the release of free flavins [11], which indicates that a deregulation of the flavin synthesis pathway seems feasible. While not generally perceived, these data indicate that *P. pastoris *can be regarded as flavinogenic at least on methanol substrates.

As *P. pastoris *is well established as a host system for production of heterologous proteins, most necessary tools for genetic manipulation are available [12]. The genome has been sequenced but still not published, so that the identification and manipulation of genes of more complex pathways has been significantly hampered. Recently the genome sequence has been made available through the commercial ERGO platform of Integrated Genomics, Inc. [13]. Thus the basis for cell and metabolic engineering of *P. pastoris *has been significantly broadened. This methodological basis as well as metabolic features like a strong pentose phosphate pathway [14] predestine *P. pastoris *as a production platform not just for proteins, but also for primary and secondary metabolites. For these reasons we have decided to employ *P. pastoris *as a model to study the riboflavin synthesis pathway in more detail.

## Methods

### Strains

*P. pastoris *X-33 (wild type), *GS115 (his4) *(both from Invitrogen, Carlsbad, CA, USA), *P. guilliermondii *DSM 70051

*Escherichia coli *Top10 (Invitrogen), *E. coli *NovaBlue (Novagen)

### DNA manipulation

If not stated otherwise, standard procedures were used for DNA manipulation. PCR was carried out with KOD XL Polymerase supplied by Novagen, according to the Novagen user protocol. For oligonucleotide primers used in this work see Table 1.

**Table 1:** Primers used for PCR amplifications

ScR1fw	GTACGGCCCAGCCGGCCACGATGACCATAGATAACTACGAC
ScR1bw	GTACGCGGCCGCTTATATTGCCAGCGTCGATG
PpR1fw	GTACGGCCCAGCCGGCCACGATGTCCGCCGCTCACGATATTTC
PpR1bw	GTACGCGGCCGCATCATTTCACCATTAC
PpR1fwPro	GTACGCGGCCGCTGGTTTGCCGTTTCATCAGC
PpR1bwPro	GTACGGATCCCGCAAGAGTGGGGCATAAAAT
PpR2fw	GTACGGCCCAGCCGGCCACGATGAGTAAAAGACTTCCACAGAGAGATGC
PpR2bw	GTACGCGGCCGCCTTTGGTTTTTCCGGCTCGTATTTG
PpR2fwPro	GTACGGATCCCTGGTAATATCTTGTAACTAATCC
PpR2bwPro	GTACGGATCCATCCCCAAGACTTCTACACTACAT
PpR3fw	GTACGGCCCAGCCGGCCACGATGTCCGTGTTTACGCCAATAGAG
PpR3bw	GTACGCGGCCGCTCTCTGTATTCCTATCCATCGTATC
PpR3fwPro	GTACGGATCCGTGGCGTGTATGAGGTAAAACTGC
PpR3bwPro	GTACGGATCCAGGGCTAATGACGGACAACTTCTT
PpR4fw	GTACGGCCCAGCCGGCCACGATGCTCATATTACGTAATAC
PpR4bw	GTACGCGGCCGCGAGGGCTCTTAACAAATAC
PpR4fwPro	GTACGGATCCGCCTCGTCACCTTCGTCATCT
PpR4bwPro	GTACGGATCCTTGCCTCACTGTGTCTTTCTTTAT
PpR5fw	GTACGGCCCAGCCGGCCACGATGTTTACGGGAATAGTGGAAAT
PpR5bw	GTACGCGGCCGCCCCTAGTTAGATATAAAATGGAG
PpR5fwPro	GTACGGATCCCCCATTGCGAGCGACCTTC
PpR5bwPro	GTACGGATCCAAATTTTTCCATCACTTATC
PpR7fw	GTACGGCCCAGCCGGCCACGATGTCATTTGTGCCCTTTCTTG
PpR7bw	GTACGCGGCCGCGTGGTCGGTATGCCAGTTATAG
PpR7fwPro	GTACGGATCCCGATCTGGGTTATTCTCTTCTTTA
PpR7bwPro	GTACGGATCCTCTTTGGACGGCGGTGTG

Sc: *S. cerevisiae*, Pp: *P. pastoris*, R1-7: *RIB1*-*RIB7 *gene, fw: forward primer, bw: backward primer, no addition signifies that this primer pair is for amplification of the complete coding sequence, addition of Pro signifies that this primer pair is for the amplification of the upstream sequence.

Transformation of *P. pastoris *by electroporation was performed following the standard protocol provided by the Pichia manuals from Invitrogen. Transformants were selected on 100 μg/mL Zeocin and 500 μg/mL Geneticin (Invitrogen) respectively.

### Cloning procedures

All the restriction enzymes, Calf Intestine Phosphatase, T4 DNA Polymerase and T4 DNA Ligase were supplied by New England Biolabs. For the overexpression of the *RIB *genes the expression vectors: pGAPZB (Invitrogen) and pGAPHIS [15] were employed. The PCR products derived from genomic *P. pastoris *X-33 DNA were amplified with the corresponding primers shown in Table 1. The expression vectors and *RIB *gene PCR products were digested with the restriction enzymes SfiI and NotI and ligated with T4 DNA Ligase to gain the desired *RIB *gene expression vectors. For the construction of the promoter replacement cassettes (PRC) the pSTBlue-1 standard cloning vector (Novagen) was used. The stepwise assembly of the PRC started with the blunt cloning of the ZeoLox cassette into the EcoRV site of pSTBlue-1. For construction of the ZeoLox cassette, the Zeocin resistance cassette was cut from pGAPZB and cloned into pUG6 [16], thereby replacing the kanMX cassette between the loxP sites. The pSTBlue_ZeoLox vector was SacI digested and blunt ended with the T4 DNA polymerase and subsequently NotI digested. This procedure gave the acceptor vector for the GAP promoter fragment including the multiple cloning site of pGAPZB (Invitrogen) which was BglII digested blunt ended and subsequently NotI digested. This cloning step gave pSTBlue_ZeoLox_GAP_MCS in which the *RIB *genes were SfiI/NotI inserted as described above. The PRC was completed by the BamHI cloning of the promoter region PCR products into the unique BamHI site upstream of the ZeoLox cassette. The *P. pastoris *clones gained by transformation with the expression vectors and the PRC are listed in Table 2. For construction of the transient Cre-recombinase expression vector pKTAC-CRE, the kanMX4 cassette from pFA6-kanMX4 [17] was cut BglII/SacI and inserted with blunt ends into the plasmid pYX022 (R&D Systems), opened AatII/KpnI, blunt ended. The resulting vector was cut with BglII, blunt ended and combined with the ARS/CEN cassette from pYC131 [18], cut FseI, blunt ended. The cre-recombinase from pSH47 [19] was inserted into the MCS of this vector. The ARS/CEN confers sufficient stability in *P. pastoris *for transient expression of the recombinase, but curing of the strains from the plasmid is easily possible by further cultivation without antibiotic.

**Table 2:** Yeast strains described in this work

Strain short name	Description
X-33	*P. pastoris *X-33 wild type (wt)
GS115	*P. pastoris *GS115 HIS^-^
X-33 *S*c*RIB1*	X-33 + pGAPZB_*ScRIB1*
X-33 *PpRIB1*	X-33 + pGAPZB_*PpRIB1*
X-33 *PpRIB2*	X-33 + pGAPZB_*PpRIB2*
X-33 *PpRIB3*	X-33 + pGAPZB_*PpRIB3*
X-33 *PpRIB4*	X-33 + pGAPZB_*PpRIB4*
X-33 *PpRIB5*	X-33 + pGAPZB_*PpRIB5*
X-33 *PpRIB7*	X-33 + pGAPZB_*PpRIB7*
GS115 *PpRIB1*	GS115 + pGAPHIS_*PpRIB1*
GS115 *PpRIB1*+empty	GS115 + pGAPHIS_*PpRIB1 *+ pGAPZB_empty
GS115 *PpRIB1*+*1*	GS115 + pGAPHIS_*PpRIB1 *+ pGAPZB_*PpRIB1*
GS115 *PpRIB1*+*2*	GS115 + pGAPHIS_*PpRIB1 *+ pGAPZB_*PpRIB2*
GS115 *PpRIB1*+*3*	GS115 + pGAPHIS_*PpRIB1 *+ pGAPZB_*PpRIB3*
GS115 *PpRIB1*+*4*	GS115 + pGAPHIS_*PpRIB1 *+ pGAPZB_*PpRIB4*
GS115 *PpRIB1*+*5*	GS115 + pGAPHIS_*PpRIB1 *+ pGAPZB_*PpRIB5*
GS115 *PpRIB1*+*7*	GS115 + pGAPHIS_*PpRIB1 *+ pGAPZB_*PpRIB7*
X-33 PRC *RIB1*	X33 + PRC_*PpRIB1*
X-33 PRC *RIB1*+*3*	X33 + PRC_*PpRIB1 *+ PRC_*PpRIB3*
X-33 PRC *RIB1*+*3*+*7*	X33 + PRC_*PpRIB1 *+ PRC_*PpRIB3*+ PRC_*PpRIB7*
X-33 PRC *RIB1*+*3*+*7*+*5*	X33 + PRC_*PpRIB1 *+ PRC_*PpRIB3*+ PRC_*PpRIB7 *+ PRC_*PpRIB5*
X-33 PRC *RIB1*+*3*+*7*+*5*+*2*	X33 + PRC_*PpRIB1 *+ PRC_*PpRIB3*+ PRC_*PpRIB7 *+ PRC_*PpRIB5 *+ PRC_*PpRIB2*
X-33 PRC *RIB1*+*3*+*7*+*5*+*2*+*4*	X33 + PRC_*PpRIB1 *+ PRC_*PpRIB3*+ PRC_*PpRIB7 *+ PRC_*PpRIB5 *+ PRC_*PpRIB2*+ PRC_*PpRIB4*

### Shake flask experiments

Shake flask experiments were carried out in 250 mL baffled shake flasks on a Multitron II shaker (Infors, Switzerland) at 28°C with 180 rpm. Defined culture medium was composed of 1.34% (w/v) Difco™ Yeast Nitrogen Base without Amino Acids (Becton, Dickinson and Company, Sparks, MD, USA), 5% (w/v) glucose, 100 mM potassium phosphate pH 6.0, 4 × 10^-5^% (w/v) biotin, 4 × 10^-3^% (w/v) L-histidine, 5 × 10^-3^% (w/v) L-glutamic acid, L-methionine, L-lysine, L-leucine, and L-isoleucine, 0,3% (w/v) CaCO_3_.

### Fed-batch fermentations

A preculture of the respective *P. pastoris *strains incubated with shaking at 28°C for 24 h on YPG (per liter: 10 g yeast extract, 10 g peptone, 10 g glycerol) was used to inoculate the starting volume (1.75 liters of batch medium) of the bioreactors to a starting optical density at 600 nm of 1.0. Fermentations were carried out in 5.0-liter working volume bioreactors (Minifors, Infors, Switzerland) with a computer-based process control. Fermentation temperature was controlled at 25°C, pH was controlled at 5.0 with addition of 25% ammonium hydroxide, and the dissolved-oxygen concentration was maintained above 20% saturation by controlling the stirrer speed between 600 and 1,200 rpm, whereas the airflow was kept constant at 100 liters h^-1^.

The batch medium contained (per liter) 2.0 g citric acid, 12.4 g (NH_4_)_2_HPO_4_, 0.022 g CaCl_2_·2H_2_O, 0.9 g KCl, 0.5 g MgSO_4_·7H_2_O, 46.5 g glycerol, and 4.6 ml PTM_1 _trace salts stock solution. The pH was adjusted to 5.0 with 25% HCl. The glucose fed-batch solution contained (per liter) 550 g glucose·1 H_2_O, 10 g KCl, 6.45 g MgSO_4_·7H_2_O, 0.35 g CaCl_2_·2H_2_O, and 12 ml PTM_1 _trace salts stock solution. The PTM_1 _trace salts stock solution contained (per liter) 6.0 g CuSO_4_·5H_2_O, 0.08 g NaI, 3.0 g MnSO_4_·H_2_O, 0.2 g Na_2_MoO_4_·2H_2_O, 0.02 g H_3_BO_3_, 0.5 g CoCl_2_, 20.0 g ZnCl_2_, 65.0 g FeSO_4_·7H_2_O, 0.2 g biotin, and 5.0 ml H_2_SO_4 _(95 to 98%). All chemicals for PTM_1 _trace salts stock solution were from Riedel-de Haën (Seelze, Germany), except for biotin (Sigma, St. Louis, MO) and H_2_SO_4 _(Merck Eurolab).

After approximately 50 h, the batch was finished and the glucose fed batch with a feed rate of 16 g h^-1 ^was started for a period of about 160 h. The cultivations were prolonged between 24 and 50 h (without feed) to analyse if further riboflavin was accumulated. Samples were taken frequently and processed as described previously in [20].

### Flow cytometry

Flow cytometric analyses were performed on a FACSCalibur instrument (Becton Dickinson, Franklin Lakes, N.J.). The cells were excited by using a 15 mW, 488 nm air-cooled argon ion laser, and the fluorescence emission was measured through a 530 ± 15 nm band-pass filter (FL1). Threshold settings were adjusted so that the cell debris were excluded from the data acquisition. A total of 10,000 cells were measured for every sample. Data analysis was performed afterwards with WinMDI 2.8 software, version 1.0 [21].

### Confocal laser scanning microscopy

Cells were grown on defined culture medium as described above. Fluorescence microscopy was performed by using a LEICA TCS SP2 microscope (excitation 488 nm, emission 494–541 nm).

### Quantification of riboflavin by HPLC

Riboflavin concentrations were determined by HPLC using a Sigma Nucleosil C18 (10 mm * 4.6 mm ID, 5 μm) guard column and a Sigma Nucleosil C18 (150 mm * 4.6 mm ID, 5 μm) column with an isocratic flow of 1 mL/min running buffer (50 mM NaH_2_PO_4_-H_3_PO_4 _pH = 3; 1 mM tetramethyl ammonium chloride; 12% acetonitrile (v/v)) [22].

Culture supernatant was achieved by centrifugation in eppendorf 2 mL tubes for 1 minute at 13,000 rpm. Prior to injection on the HPLC column the samples were mixed with a 2-times concentrated running buffer in an equal ratio and filtered over PVDF Durapore Syringe Driven Filter Unit (Millipore) with a pore size of 0.22 μm. 100 μL of the final samples were loaded on the HPLC column and quantified by the peak height of absorption, detected at 223 nm and 445 nm respectively.

## Results

### A single gene overexpression renders *P. pastoris *flavinogenic

Previously published research on the formation of riboflavin in different riboflavin overproducing organisms indicates that the enzymatic activity of GTP-cyclohydrolase II (*RIB1*, Figure 1) plays a key regulatory role in the riboflavin biosynthetic pathway. Consequently, the overexpression of this gene was the first step for deregulation of riboflavin biosynthesis in *P. pastoris*. Since initially, the genome sequence of this yeast was not available to us we decided to express the GTP-cyclohydrolase II gene of *S. cerevisiae *(*ScRIB1*) [23].

**Figure 2:**
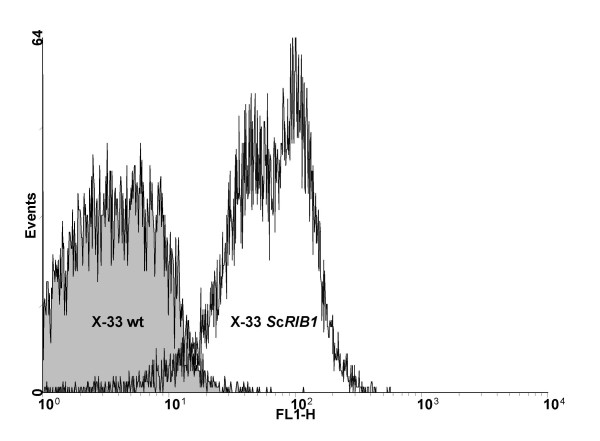
**Flow cytometric analysis of *P. pastoris***. The histogram displays the number of cells with the corresponding fluorescence at 530 nm (FL1). The strain overexpressing the *ScRIB1 *gene has an increased autofluorescence, indicating a higher intracellular riboflavin concentration.

The respective gene was PCR-amplified from genomic DNA and cloned into the *P. pastoris *expression vector pGAPZB, from where the gene is expressed under control of the constitutive glyceraldehyde-3-phosphate dehydrogenase promoter (P_*GAP*_). Transformation of *P. pastoris *X-33 led to yellow colonies, clearly indicating the accumulation of a flavin. Furthermore, flow cytometric analysis revealed a significantly increased (auto-) fluorescence signal of the clones expressing *ScRIB1 *in comparison to the wild type (wt) strain (Figure 2). The wavelength of FL1 (530 nm ± 15 nm) corresponds to the fluorescent emission of riboflavin pointing to an increased riboflavin content of the cells. Intracellular riboflavin is localized in the vacuole (data not shown), as previously described for *A. gossypii *[24].

The production of riboflavin of wt *P. pastoris *X-33 and X-33 *S*c*RIB1 *was compared to the flavinogenic yeast *P. guilliermondii *in shake flasks cultures using minimal medium and glucose as carbon source. Riboflavin was proven to be stable under these conditions in presence of yeast cells for at least 10 days (data not shown). While the wt *P. pastoris *X-33 did not produce any significant amount of riboflavin, X-33 *S*c*RIB1 *accumulated 4 mg/L and *P. guilliermondii *produced 8 mg/L of riboflavin. This result clearly indicates that the single gene overexpression of *RIB1 *changed the phenotype of *P. pastoris *to flavin overproduction. Interestingly, both, *P. pastoris *X-33 *ScRIB1 *and *P. guilliermondii *accumulated riboflavin in the culture supernatant in the late stationary phase, when the glucose in the culture broth was completely consumed. Hence, flavin production is at least partly uncoupled from growth in the naturally flavinogenic yeast as well as the recombinant *P. pastoris *strain.

In order to test whether the upstream pathways of riboflavin synthesis, particularly the provision of GTP, are the next rate limiting steps, precursors for GTP synthesis (as glycine, threonine, and glutamine [2]) were fed to shake flask cultures of *P. pastoris *X-33 *ScRIB1*. However, no positive impact on riboflavin synthesis could be detected. Similarly, UV mutagenesis followed by selection on deoxyglucose (for a deregulated glycolytic flux or uptake) or tubercidin (7-deaza-adenosine, for deregulated GTP formation) did not yield any positive effect on riboflavin production. These approaches have been successfully applied to enhance riboflavin synthesis in *A. gossypii *[2] and *Candida famata *[25]. The fact that they do not have an effect on this *P. pastoris *strain indicates that further limiting step(s) have to be found downstream, within the riboflavin pathway itself. However, the data do not allow the conclusion that GTP synthesis would not limit riboflavin production at all, especially at higher rates.

### Identification of further key players within the *P. pastoris *riboflavin biosynthetic pathway

Five known enzymes are located downstream of *RIB1 *(Figure 1), being potentially limiting for riboflavin production. Individual overexpression of each of these genes was examined in view of riboflavin production of the resulting recombinant strain. Since the *P. pastoris *genome sequence became available to us, we decided to proceed with the overexpression of the homologous *P. pastoris *genes instead of heterologous genes from *S. cerevisiae*. All *RIB *genes from *P. pastoris *were PCR-amplified from genomic DNA and cloned into the *P. pastoris *expression vector pGAPZB. Expression of *PpRIB1 *in *P. pastoris *X-33 led to yellow colonies as has been noted for overexpression of *ScRIB1*. However, none of the other genes showed any effect concerning neither the colony color nor the production of riboflavin in shake flask cultures when overexpressed from pGAPZB in *P. pastoris *X-33. This indicates that constitutive overexpression of *RIB1 *is an essential prerequisite for riboflavin overproduction in *P. pastoris*.

Consequently, for the identification of further pacemaker enzymes within the pathway, the other *RIB *genes had to be individually co-expressed in combination with *RIB1*. *PpRIB1 *was cloned into the GAPHIS expression vector and integrated into the *HIS4 *locus of a *P. pastoris *GS115 strain. The riboflavin production of the *P. pastoris *GS115 *PpRIB1 *was tested in shake flask cultures and an equal production of riboflavin compared to X-33 *PpRIB1 *was observed (Table 3). GS115 *PpRIB1 *was further transformed with the other *RIB *genes in pGAPZ, resulting in the strains: GS115 *PpRIB1*+empty, GS115 *PpRIB1*+*1*, GS115 *PpRIB1*+*2*, GS115 *PpRIB1*+*3*, GS115 *PpRIB1*+*4*, GS115 *PpRIB1*+*5*, and GS115 *PpRIB1*+*7*.

**Table 3:** Riboflavin accumulation of *P. pastoris *strains, overexpressing two *RIB *genes

Strain	Riboflavin in supernatant
X-33 *ScRIB1*	3.9 mg/L +/- 0.25
GS115 *PpRIB1*	3.0 mg/L +/- 0.10
GS115 *PpRIB1*+empty	3.0 mg/L +/- 0.18
GS115 *PpRIB1+1*	7.1 mg/L +/- 0.04
GS115 *PpRIB1+3*	10.1 mg/L +/- 0.24
GS115 *PpRIB1+7*	4.9 mg/L +/- 0.19

The cells were cultivated in shake flasks on defined medium and glucose as carbon source. Shake flask experiments and HPLC measurements were run in duplicates. Mean values +/- standard deviations are given.

A combination of *RIB1 *with *RIB3*, *RIB7 *or a second copy of *RIB1 *enhances the riboflavin production in shake flask experiments as shown in Table 3. While a strain with a single copy of *RIB1 *accumulates 3 mg/L of riboflavin, a strain overexpressing *RIB1 *and *RIB7 *accumulates 5 mg/L. Two copies of *RIB1 *lead to an accumulation of 7 mg/L and the combination of *RIB1 *with *RIB3 *results in an accumulation of 10 mg/L of riboflavin, which is more than *P. guilliermondii *accumulates under comparable conditions. Co-expression of any other gene from the riboflavin pathway (*RIB2*, *RIB4 *or *RIB5*, coding for the last enzymes of the metabolic pathway) did not show any increase of riboflavin production. The bottleneck for riboflavin production appears therefore to be in the beginning of the pathway as the first three genes show an impact on final product concentration, but not the genes located in the final part of the pathway.

It is interesting to note that the addition of a second copy of *RIB1 *leads to a significantly more intense yellow color of the colonies compared to a strain overexpressing only one copy of *RIB1*. This is in contrast to colonies of a strain combining *RIB1 *and *RIB3*, which produces even more riboflavin in shake flask cultures then the double *RIB1 *strain. The addition of *RIB3 *does not change the color of the colonies significantly. However, the agar around the colonies becomes intensely yellow colored. This phenomenon does not occur for any of the other strains. It appears therefore, that the double *RIB1 *clones accumulate more riboflavin intracellularly, whereas, the riboflavin from the *RIB1*/*RIB3 *clones diffuses more efficiently into the agar. We have currently no explanation, which role *RIB3 *plays for diffusion or export of riboflavin. Further research has to be dedicated to this question.

The next step for the construction of an improved riboflavin producing *P. pastoris *strain was the combination of the early genes of the pathway and subsequently to test if the late genes have any impact at all for riboflavin production in *P. pastoris*.

### Targeting the *RIB *genes at the native locus

If conventional techniques are to be used, six markers are required for the overexpression of six different genes – as was the goal of this work. However, *P. pastoris *strains carrying six auxotrophies have not been constructed yet and the number of possible dominant markers is also limited. Consequently, only the rescue and repeated use of the same marker allows the introduction of a higher number of recombinant DNA constructs. The use of the cre/lox system allows the easy rescue of any desired marker [26] and [27]. However, a disadvantage is that the constructs have to be introduced with sufficient distance into the genome to make sure that the lox recombination eliminates the desired marker cassette but does not destabilize other parts of the genome.

**Figure 3:**
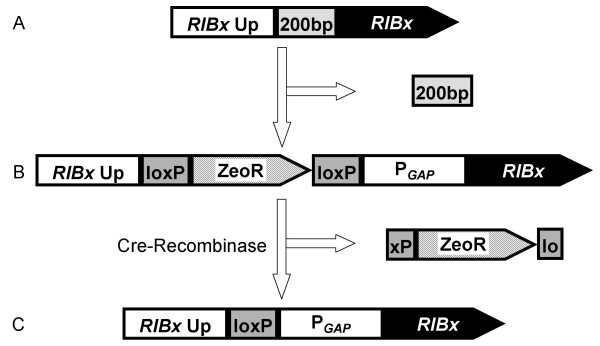
**Schematic representation of the exchange of the native *RIB *promoters with a promoter replacement cassette (PRC)**. (A) depicts the native *RIB *locus. By homologous recombination the PRC (B) is inserted into the locus thereby deleting 200 bp upstream of the coding sequence. Transient expression of the Cre-recombinase leads to the excision of the Zeocin marker cassette (ZeoR), resulting in (C). In consequence the *RIB *gene is expressed in its native locus under the control of the constitutive glyceraldehyde-3-phosphate dehydrogenase promoter (P_GAP_) and no marker remains with the construct. *RIBx *Up: 5' untranslated region upstream of the corresponding *RIB *gene.

In order to fulfill these requirements we opted for a replacement of the native promoter of every single *RIB *gene with the strong and constitutive P_*GAP *_of *P. pastoris*, instead of introducing further copies of the genes. Figure 3 shows a schematic representation of the respective promoter replacement cassettes (PRC). The cassettes comprise 500 bp of homologous sequence on both sides to allow for targeted integration into the genome. Integration of the cassettes leads to a deletion of 200 bp directly upstream of the ATG, which putatively comprise the core elements of the native promoters. These 200 bp are replaced with the Zeocin resistance cassette flanked by two loxP sites and the desired P_*GAP *_promoter element, providing a strong constitutive expression of the *RIB *gene.

Transformation of the *RIB1 *PRC into *P. pastoris *X-33 changed the colony color to yellow like it was observed for all strains overexpressing *ScRIB1 *or *PpRIB1*. Marker removal by transient expression of the Cre-recombinase allowed the next round of transformation to target the next gene. An interesting notion relates to the screening of a first round of clones that had been transformed with a cassette comprising the complete *RIB1 *coding sequence and not only the first 500 bp. It turned out that the yellow color of the selected colonies was significantly reduced after marker rescue. By PCR we proved that the clones with the most intense yellow color integrated the expression cassette more than one time. The cre/lox recombination eliminated these cassettes and only one remained. By using only 500 bp of the coding sequence for the recombination cassettes we made sure that even if multiple cassettes are introduced by the first transformation only one functional copy of the gene is generated, avoiding any phenotypic difference of the clones before and after cre/lox recombination.

### Stepwise deregulation of the entire riboflavin pathway

Table 4 shows the results of shake flask cultures of the *P. pastoris *strains with sequential deregulation of the *RIB *genes. As expected, the overexpression of only *RIB1 *leads already to a significant accumulation of riboflavin in the culture supernatant (7 mg/L). X-33 PRC *RIB1+3 *and X-33 PRC *RIB1+3+7 *produce 9 mg/L or 10 mg/L respectively. As presumed the addition of *RIB3 *leads to an increased accumulation of the vitamin, but subsequent addition of *RIB7 *appears to have only a minor effect.

**Table 4:** Riboflavin accumulation of *P. pastoris *strains, with an increasing number of deregulated *RIB *genes

Strain	Riboflavin in supernatant
X-33 PRC *RIB1*	6.9 mg/L +/- 0.17
X-33 PRC *RIB1+3*	9.1 mg/L +/- 0.25
X-33 PRC *RIB1+3+7*	10.2 mg/L +/- 0.17
X-33 PRC *RIB1+3+7+5*	13.2 mg/L +/- 0.47
X-33 PRC *RIB1+3+7+5+2*	14.9 mg/L +/- 0.76
X-33 PRC *RIB1+3+7+5+2+4*	20.2 mg/L +/- 0.83

The cells were cultivated in shake flasks on defined medium and glucose as carbon source. Shake flask experiments and HPLC measurements were run in duplicates. Mean values +/- standard deviations are given.

To avoid possible metabolic problems caused by the accumulation of intermediate products of the riboflavin synthesis we decided to increase the catalytic turnover of the last enzyme in the pathway as the following step. The resulting strain X-33 PRC *RIB1+3+7+5 *accumulates 13 mg/L riboflavin. Addition of *RIB2 *has a further slightly positive effect (15 mg/L) and closing the last gap by further overexpression of *RIB4 *has the most pronounced effect on riboflavin accumulation. The strain X-33 PRC *RIB1+3+7+5+2+4 *accumulates 20 mg/L of riboflavin in shake flask cultures.

While product accumulation is steadily increasing with the addition of further genes, the autofluorescence of the cells measured by flow cytometry is not (data not shown). This indicates that the amount of riboflavin retained inside of the cells reaches an upper limit and any further produced vitamin is secreted (or diffusing) into the culture supernatant.

The sequential increase of riboflavin accumulation with the addition of further *RIB *genes shows clearly that the regulation of riboflavin production is distributed over and dependent on more genes and enzymes of the entire pathway and not on just one single pacemaker enzyme alone. *RIB1 *is the first bottleneck – without increase of *RIB1 *no increase in the flux is possible. However, at least *RIB3*, *RIB5*, *RIB2 *and *RIB4 *play significant roles in controlling the pathway too. Addition of glycine, a precursor for GTP has no or only a slight impact on riboflavin production of all strains (data not shown), indicating that still the pathway itself is limiting and not the supply of precursors.

**Figure 4:**
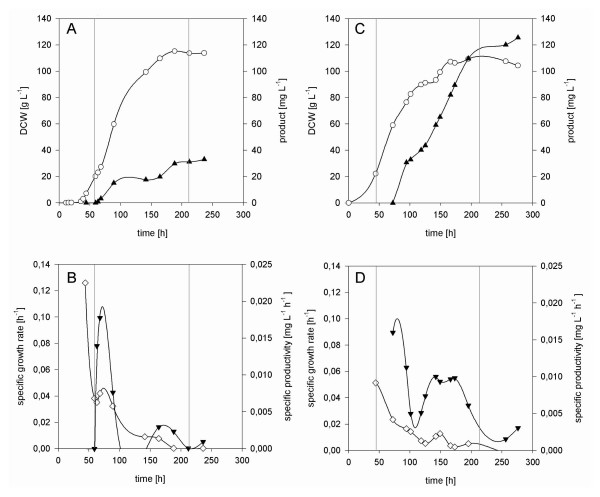
**Kinetics of standard fed batch cultures of *P. pastoris *overexpressing the *RIB1 *gene (A and B) or all *RIB *genes (C and D), respectively**. The yeasts were cultivated on mineral medium with glucose as carbon source. Open circles represent dry cell weight (DCW), closed triangles indicate the riboflavin concentration (A and C). Open diamonds signify the specific growth rate and closed triangles (tip down) represent the specific productivity (B and D). Grey lines divide the three phases of the cultivations, namely the batch phase in the beginning, the central fed-batch phase and the stationary phase in the end.

To assess the future potential of the constructed strains and to verify that the trend seen in the shake flask experiments holds true also for high cell density cultivations, the strains X-33 PRC *RIB1 *and X-33 PRC *RIB1+3+7+5+2+4 *were grown in fed-batch mode in bioreactors. Figure 4 shows the corresponding results. While the wt strain does not accumulate riboflavin in high cell density cultures grown on glucose as carbon source (data not shown) the strain overexpressing only *RIB1 *accumulates 33 mg/L in the culture broth, which corresponds to 50 mg/L in the supernatant. (The difference is due to the volume of the cells, which cannot be neglected in high cell density cultures). The strain overexpressing all of the genes from the riboflavin biosynthetic pathway (X-33 PRC *RIB1+3+7+5+2+4*) accumulates under the same conditions more than 125 mg/L in the culture broth, which corresponds to 175 mg/L in the supernatant. Confirming the indications from shake flask experiments a major part of the riboflavin accumulation takes place towards the end of the cultures, when the growth rate of the cells is already significantly reduced. Riboflavin accumulation is therefore at least partly uncoupled from growth. This is particularly surprising as the promoter driving the expression of the genes is most active in growing cells as is the supply of the precursors GTP and ribulose-5-phosphate.

## Discussion

Having established that *P. pastoris *can be modified into a flavin producer by overexpression of *ScRIB1*, we identified and cloned the respective *RIB *gene homologs of *P. pastoris*. As the feeding of precursers of the pathway did not enhance the production of riboflavin it was obvious that further regulation should be found further downstream of *RIB1*. By overexpression of all single genes alone and combined it was established that all of them have a regulatory impact, while *RIB1 *is an essential pacemaker of the pathway.

*RIB3 *and *RIB7*, as the next early steps of both branches of the pathway, could further enhance riboflavin synthesis when co-overexpressed with *RIB1*, while the late steps of the pathway did not have an impact together with *RIB1*, which indicates that the control of *RIB3 *and *RIB7 *limits the flux so that the deregulation of the late genes cannot become effective. Therefore it was obvious to co-overexpress all of the *RIB *genes under control of a constitutive promoter. Instead of cloning the *RIB *genes, they were overexpressed by exchanging their native promoters to the GAP promoter by homologous recombination. Stepwise deregulation of all six *RIB *genes lead to a strain producing 175 mg/L, 3.5 times more than the *RIB1 *strain, in fed batch cultures. Interestingly, riboflavin production was not entirely growth associated, but was high also at rather low specific growth rates towards the end of the cultures. Riboflavin production in *A. gossypii *was shown to occur mainly in the late growth phase/stationary phase [28]. It is interesting that this growth decoupling is also observed when all *RIB *genes are expressed under the GAP promoter, which has been shown to be strictly growth coupled in *P. pastoris *both for the native GAP gene and heterologous genes (Maurer, Gasser und Mattanovich unpublished). As the synthesis of the metabolic precursors GTP and ribulose-5-phosphate will be growth related too, there is no obvious explanation for the growth decoupling of the riboflavin pathway. It may be speculated that a higher turnover of GTP into RNA and ribulose-5-phosphate in the pentose phosphate pathway may lead to a reduction of the riboflavin pathway flux at higher specific growth rates. While this work has concentrated on deregulation of *RIB *gene expression, one has to consider that the riboflavin synthesis enzymes are likely to be regulated also by feedback inhibition. Compartmentalization of precursor synthesis and riboflavin release may constitute another bottleneck. However, more research will be necessary to establish these relations.

## Conclusion

The basis for understanding the regulation patterns of the riboflavin pathway in *P. pastoris *has been achieved by this study. This work can serve as a starting point to establish strains for riboflavin production, as well as engineered strains for the high level expression of flavin containing enzymes.

## Competing interests

The authors declare that they have no competing interests.

## Authors' contributions

HM, DM and MS participated in the design of the study, analysis of the data and wrote the paper. HM performed the experimential part of the work.

All authors have read and approved the final version of the manuscript.
